# On the statistical performance of Granger-causal connectivity estimators

**DOI:** 10.1007/s40708-015-0015-1

**Published:** 2015-04-22

**Authors:** Koichi Sameshima, Daniel Y. Takahashi, Luiz A. Baccalá

**Affiliations:** 1 Radiology & Oncology Department, Faculdade de Medicina, University of São Paulo, São Paulo, SP 01246-903 Brazil; 2 Psychology Department, Neuroscience Institute, Princeton University, Princeton, NJ USA; 3 Department of Telecommunications and Control, Escola Politécnica, University of São Paulo, São Paulo, SP Brazil

**Keywords:** Partial directed coherence, Directed transfer function, Granger causality, Null hypothesis test performance, Conditional multivariate Granger causality

## Abstract

In this article, we extend the statistical detection performance evaluation of linear connectivity from Sameshima et al. (in: Slezak et al. (eds.) Lecture Notes in Computer Science, 2014) via brand new Monte Carlo simulations of three widely used toy models under different data record lengths for a classic time domain multivariate Granger causality test, information partial directed coherence, information directed transfer function, and include conditional multivariate Granger causality whose behaviour was found to be *anomalous*.

## Introduction

This paper compares the statistical performance of linear connectivity detection [1] using four popular neural connectivity estimators. In addition to the classic Granger causality test (GCT) from [2], we employ our recently derived rigorous results [3, 4] about the asymptotic behaviour of information PDC (*i*PDC) and information DTF (*i*DTF) [5] that, respectively, generalize partial directed coherence (PDC) [6] and directed transfer function (DTF) [7] which correctly describe coupling effect size issues. A fourth method was included in this extended version of [8] and consists of the proposal put forward by [9] (*c*MVGC) for detecting conditional Granger causality between time series pairs and is applied here using their published MVGC package. There have been many recent papers [10–14] aimed at comparing contending connectivity estimation procedures. In fact, almost every new connectivity estimation procedure sports some form of appraisal by counting the number of correct detection decisions. What sets the present effort apart, as emphasized by using the word *statistical*, is that we focus on methods that have rigourous theoretically derived asymptotic detection criteria.

In the comparisons, we used Monte Carlo simulations of three widely used toy models from the literature and verified the performance of null connectivity hypothesis rejection as a function of data record length, * K*. To complement the study, we also computed false positive (FP) and false negative (FN) test rates for each estimator alternative. In the MVGC package case, false detection rates were computed with and without author-recommended corrections [9].

## Methods and results

### Monte Carlo simulations

Following our recently proposed information PDC and information DTF [5], and their corresponding rigorous asymptotic statistics (see [3] and [4] for details), we first gauged their statistical performance against that of the well-established time-domain GCT test [2]. For added comparison here, we added results from conditional connectivity detection obtained via the MVGC package [9]. Monte Carlo simulations were performed in the MATLAB environment using its normally distributed pseudorandom number generator to simulate systems with uncorrelated zero mean and unit variance innovation noise as model inputs. To test the performance of the latter four connectivity estimators, for each toy model and at each data record length, we selected values of * K* = {100, 200, 500, 1000, 2000, 5000, 10000} repeating 1000 simulations for each case. For each simulation, a burn-in set of 5000 initial data points were discarded to eliminate possible transients before selecting the* K* value of interest. We used the Nuttall-Strand algorithm for multivariate autoregressive (MAR) model estimation and the Akaike information criterion (AIC) for model order selection [15] for GCT, *i*PDC and *i*DTF, while the Levinson–Wiggins–Robinson solution of the multivariate Yule-Walker equations was used as is default for *c*MVGC [9]. Detection threshold was set in compliance to $\alpha =1\,\%$. For *i*PDC and *i*DTF, * p* values were computed at 32 uniformly separated normalized frequency points covering the whole interval with a connection being deemed detected for a given pair of structures if its * p* value resulted to be less than $\alpha$ for some frequency within the interval. This connectivity decision criterion is somewhat lax and tends to overestimate the presence of connectivity for *i*PDC and *i*DTF. In particular for *i*PDC, one should expect connectivity detection more often than GCT, i.e. more FPs are likely.

The reader may access our open MATLAB codes for GCT and for both *i*PDC and *i*DTF asymptotic statistics used in this study at http://www.lcs.poli.usp.br/~baccala/BIHExtension2014/.

The Web site, furthermore, contains the datasets of the employed simulation results and a copy of the exact version of the MVGC package used in the present comparisons [9]. This allows full reader accessing disclosure of the data/procedures with the possibility of cross-checking and replaying all results. Additional graphs and results are available there and may be consulted for details; only the overall representative behaviour is summed up here.

Next we describe the toy models and the allied simulations results.

### Model 1: Closed-loop model

**Fig. 1:**
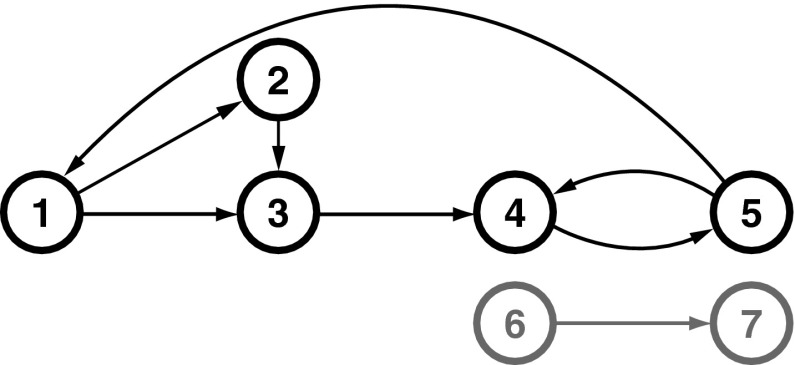
Diagram depicting the essential elements of Model 1 represented by Eq. 1 from [16]. The elements from $x_1$ to $x_5$ establish closed-loop connections, with short and long connected paths, while $x_6$ and $x_7$ are part of a completely separate substructure, i.e. disconnected from $\{x_1,\ldots ,x_5\}$, but sharing a common frequency of oscillation

It is an $\{N=7\}$-variable model, borrowed from [16] (Fig. 1), with two completely disconnected substructures, {$x_1,\,x_2,\,x_3, \, x_4, \, x_5$} and {$x_6$, $x_7$}, which share a common frequency of oscillation. The set of descriptive equations is

1 $$\begin{aligned} \left\{ \begin{aligned} x_{1}(t) &=\ 0.95\sqrt{2}x_{1}(t-1) - 0.9025x_{1}(t-2) \\&\quad+\, 0.5x_{5}(t-2)+ w_{1}(t) \\ x_{2}(t) &=-0 .5x_{1}(t-1) + w_{2}(t)\\ x_{3}(t) &=\ 0.2x_1(t-1)+ 0.4 x_{2}(t-2) + w_{3}(t)\\ x_{4}(t) &=-0.5x_{3}(t-1) + 0.25\sqrt{2}x_{4}(t-1)\\&\quad+ \,0.25\sqrt{2}x_{5}(t-1) + w_{4}(t) \\ x_{5}(t) &=-0.25\sqrt{2}x_{4}(t-1) + 0.25\sqrt{2}x_{5}(t-1)+ w_{5}(t)\\ x_{6}(t) &=\ 0.95\sqrt{2}x_{6}(t-1) - 0.9025x_{6}(t-2) + w_{6}(t) \\ x_{7}(t) &=-0.1x_{6}(t-2) + w_{7}(t), \end{aligned} \right. \end{aligned}$$

where $w_{i}$ stand for uncorrelated ${\mathcal{N}}(0,1)$ Gaussian innovations.

**Fig. 2:**
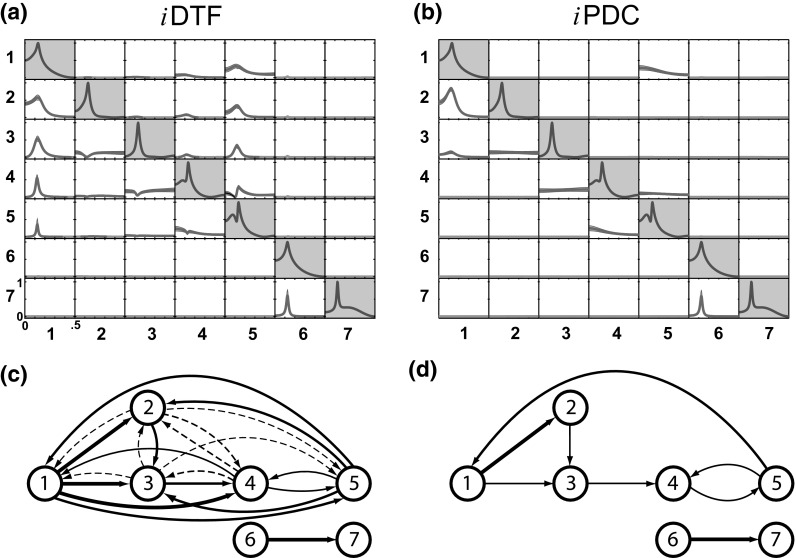
A single-trial results of **a** *i*DTF and **b** *i*PDC estimations obtained using a data simulation of Model 1 with * K* = 2000 points. In both subfigures, **a**, **b**, the main diagonal subplots with *gray background* contain power spectra, while each off-diagonal subplot represents *i*DTF or *i*PDC measure in the frequency domain with variables in *columns* representing the sources and in rows the target structures, in which significant measure is drawn in * red lines* at $\alpha = 1\,\%$, and in * green lines* otherwise. **c** Note that, as theoretically expected, according to *i*DTF estimation, all nodes of $\{ x_1,\, x_2,\, x_3,\,x_4,\,x_5\}$ set can reach one another, **d** while *i*PDC correctly exposes, similar to GCT, the immediate adjacent node’s connectivity pattern. See further discussion about the contrast between *i*DTF and *i*PDC in [17]. (Color figure online)

 Results from a single-trial example of *i*DTF and *i*PDC connectivity estimations in the frequency domain are depicted in Fig. 2a, b, respectively, with significant values, at $\alpha =0.01$, represented by red solid lines. The corresponding connectivity graph diagrams are contained in Fig. 2c, d, where arrow thickness represents estimate magnitude. Note that *i*PDC reflects adjacent connections, Fig. 2b, d, while *i*DTF, Fig. 2a, c, represents graph reachability aspects of the directed structure [17, 18]. The notion of reachability refers to the net influence from a time series onto another through various signal pathways, i.e. it measures how much of one series ends up influencing another.

#### Granger causality test for Model 1

**Fig. 3:**
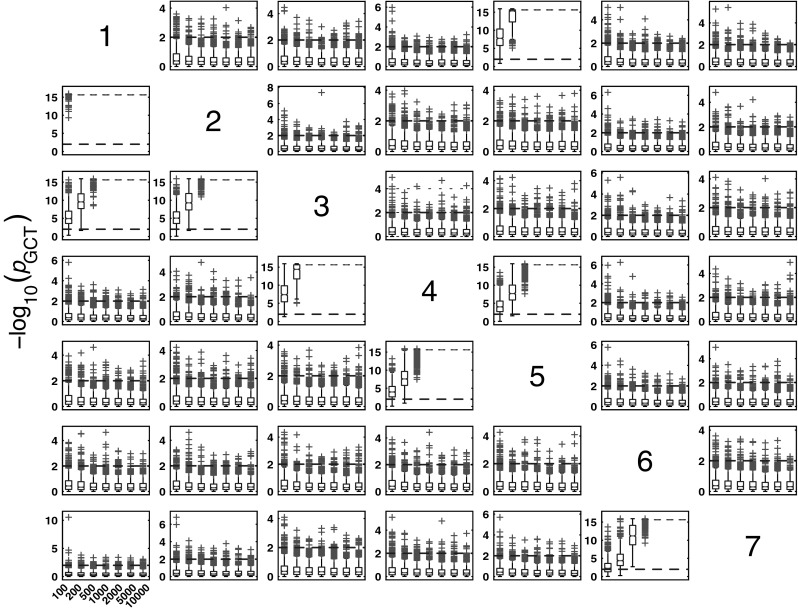
The patterns (in this and all the figures of similar kind that follow) containing subplots with variables in columns representing the sources and the target structures in rows. Each subplot possesses boxplots of the distribution of GCT −log_10_(*p* value) for 1000 Monte Carlo simulations over different record lengths * K* = {100, 200, 500, 1000, 2000, 5000, 10000} along the * x*-axis of each subplot. Since $\alpha =0.01$, values above 2 (*dashed-line*) indicate rejection of the null hypothesis of connectivity absence. *Red crosses* indicate * p* value distribution outliers, and those above *dashed-line* represent false positives (FPs) for nonexisting connections. (Color figure online)

The boxplots of −log_10_(*p* value) in Fig. 3 summarize GCT performance for Model 1 and * K* = {100, 200, 500, 1000, 2000, 5000, 10000} data record lengths. As expected, for * K* > 200, it properly detects connectivity between adjacent structures with zero observed FNs for all pairs of existing connections.

#### *i*PDC performance for Model 1

**Fig. 4:**
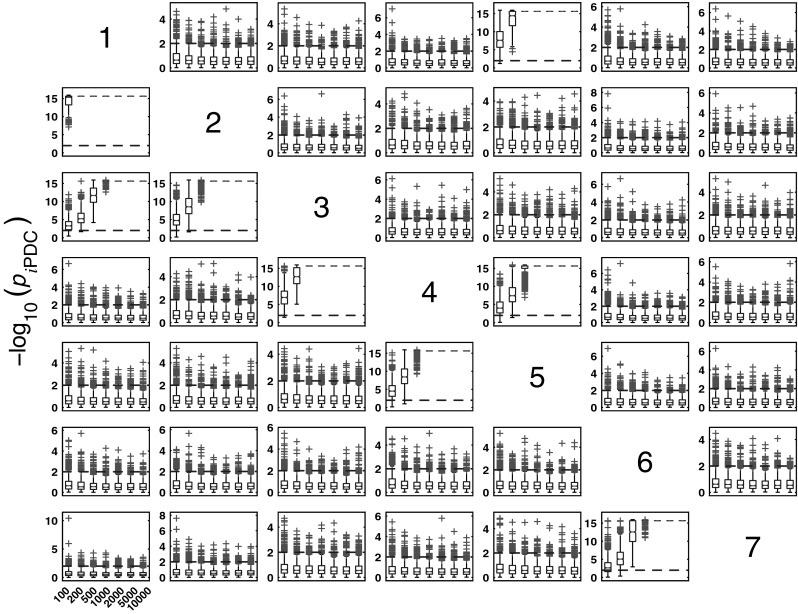
Model 1 boxplot performance summary of *i*PDC asymptotics. Most outliers for absent connections are above the $\alpha$ threshold decision line

**Fig. 5:**
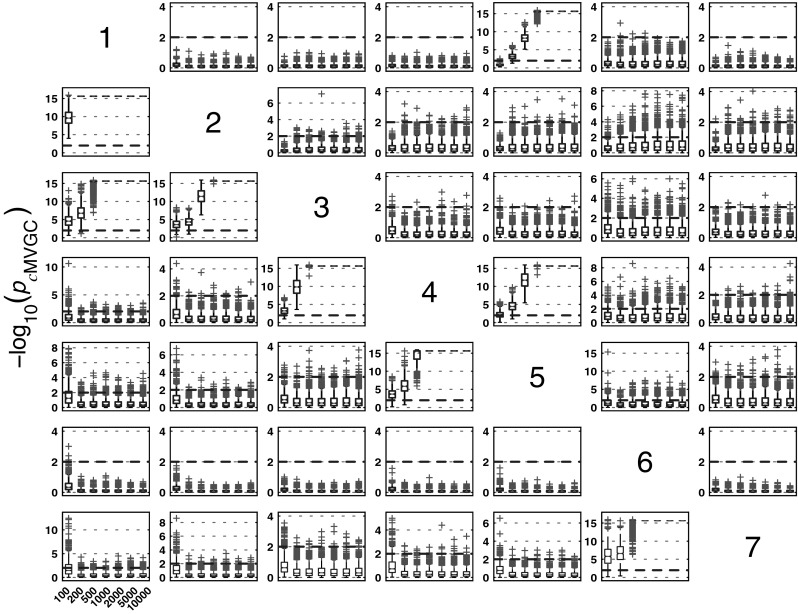
Model 1 boxplot performance summary of *c*MVGC asymptotics. Note that *red cross* outliers for the connections into $x_1$ and $x_6$ (see the * first* and * sixth rows* of subplots’ layouts) are consistently below −log_10_(*p* value) = 2, and those from $x_6$ consistently above for all $K$, something that is reflected in Fig. 8. (Color figure online)

**Fig. 6:**
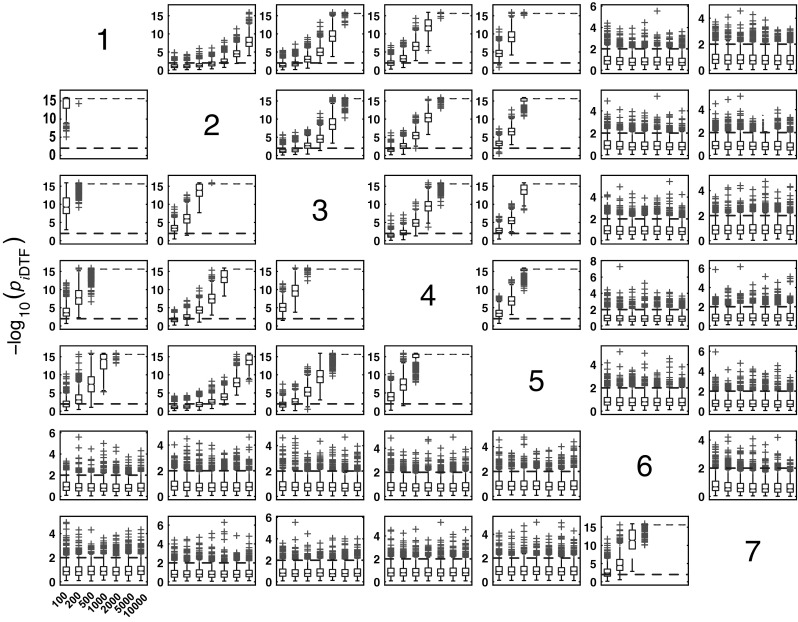
Model 1 boxplot performance summary of *i*DTF asymptotics. Note that every node of $\{ x_1,x_2,x_3,x_4,x_5\}$ set can directionally reach one another, as depicted in Fig. 2c. Note also that FN rates decrease consistently for larger * K*

**Fig. 7:**
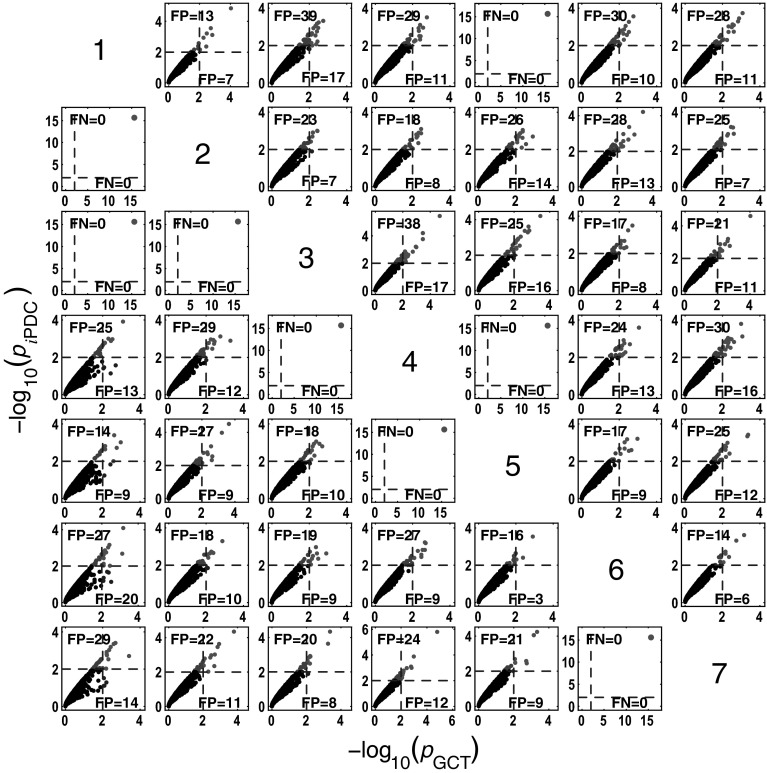
GCT and *i*PDC connectivity comparative detection performance (*K* = 2000) where *i*PDC −log_10 _(*p* value) for each one of the 1000 simulations is plotted against its GCT’s −log_10_ (*p* value). Results for all connections are clustered slightly above the 45° line with $b=1.1157 \pm 0.0283$ (median$=1.1216$; minimum$=1.0198$; and maximum$=1.1486$). The number of FP and FN detections over the 1000 simulations are also shown for *i*PDC (*top left*) and GCT (*bottom right*) for each subplot

Figure 4 summarizes the asymptotic *i*PDC statistical performances for the same data and record lengths as for GCT in Fig. 3 with similar performance (Figs. 5, 6). Closer comparison on identical trials for each estimator leads to Fig. 7 depicting *i*PDC versus GCT performance (*K* = 2000), further revealing a pattern of consistently higher FP values for *i*PDC expectedly resulting from how the test was performed with *i*PDC decision dictated by a single maximum frequency above threshold. In Fig. 7, the average slopes are above 45° consistent with the larger number of FPs for *i*PDC.

At this point, one should note that for trial-by-trial comparisons between methods only those against GCT are present for the sake of conciseness. Pairwise behaviour for other pairs of methods is easy to infer. GCT’s choice as a reference was dictated by its canonical behaviour in terms of the expected performance in the Neyman–Pearson hypothesis testing framework. In the Web site, it is possible to use available routines to examine the results that apply to the comparison between other pairs of methods.

#### *c*MVGC behaviour for Model 1

**Fig. 8:**
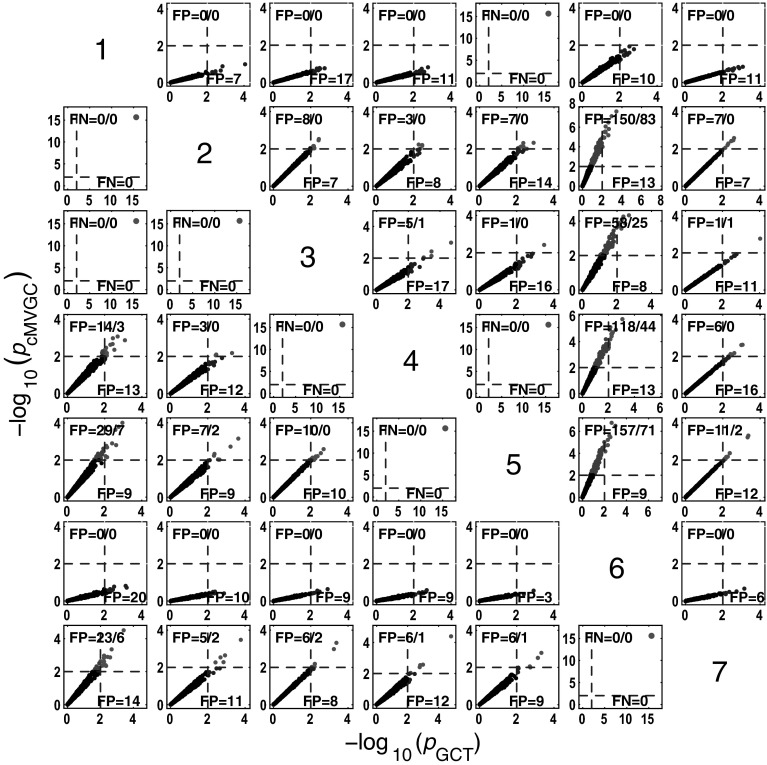
*c*MVGC versus GCT performance (*K* = 2000) clusters along lines, with high $b=0.8329 \pm 0.5741$ coefficient spread (median $=0.8417$; minimum $=0.1762$; and maximum $=2.3323$), confirming *c*MVGC’s abnomal behaviour. Connections out of $x_6$ are consistently clustered above the 45° line, which contrasts with those reaching the $x_1$ and $x_6$ consistently with low *c*MVGC FP values (*top left*) (except for $x_5 \rightarrow x_1$). *c*MVGC abnormality is apparent as FP values differ much from 10 as should happen for $\alpha =1\,\%$. *c*MVGC-corrected FPs (separated by * slash*) do not improve the situation

Figure 5 summarizes the performance of pairwise conditional MVGC in the form of boxplots. They asymptotically capture the structure of Fig. 1 despite differences compared to GCT and *i*PDC. These differences are easier to appreciate on the trial-by-trial comparison with respect to GCT (Fig. 8), which shows that *c*MVGC’s FP rates are sometimes well below the imposed $\alpha =1\,\%$ and even become more extreme after authors’ recommended corrections [9] (*K* = 2000). Note how point distributions in Fig. 8 hardly ever cluster round the 45° line for connections reaching the $x_1$ and $x_6$ oscillators. For connections leaving $x_6$, the pattern is reversed. It is this failure to meet the preset $\alpha =1\,\%$ irrespective of which connection is under consideration, which we call *anomalous* here.

#### *i*DTF performance for Model 1

Figure 6 summarizes the performance of the asymptotic statistics for *i*DTF. The boxplots clearly show that for larger sample sizes, *i*DTF correctly detects the reachability structure shown in Fig. 2c. Note that the weakest, and in this case, the farthest connection ($x_2 \rightarrow x_1$) requires longer record lengths for proper detection.

### Model 2: Five-variable model

**Fig. 9:**
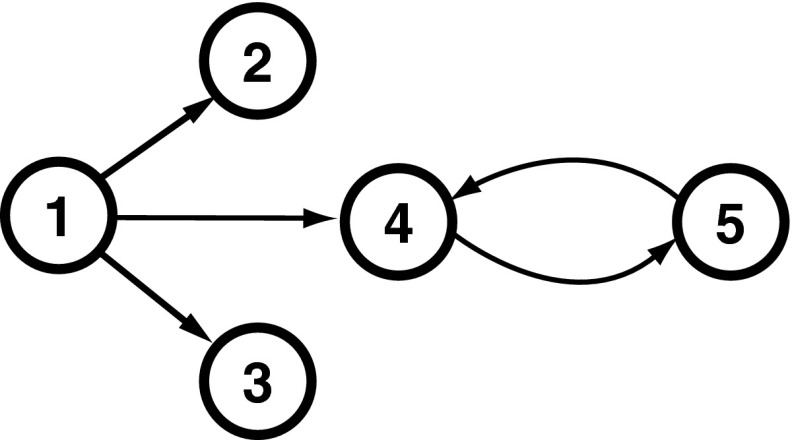
Diagram depicting the essential elements of Model 2 introduced by [6]

Model 2 introduced by [6] is graphically represented in Fig. 9 with its corresponding set of defining equations:

2 $$\begin{aligned} \left\{ \begin{aligned} x_{1}(t) &=\ 0.95\sqrt{2}x_{1}(t-1) - 0.9025x_{1}(t-2) + w_{1}(t) \\ x_{2}(t) &=\ 0.5x_{1}(t-2) + w_{2}(t) \\ x_{3}(t) &=-0.4x_{1}(t-3) + w_{3}(t) \\ x_{4}(t) &=-0.5x_{1}(t-2) + 0.25\sqrt{2}x_{4}(t-1) \\&\quad +\, 0.25\sqrt{2}x_{5}(t-1) + w_{4}(t)\\ x_{5}(t) &=-0.25\sqrt{2}x_{4}(t-1) + 0.25\sqrt{2}x_{5}(t-1) + w_{5}(t) , \end{aligned} \right. \end{aligned}$$

where $w_i$, as before, stand for uncorrelated Gaussian innovations. Computations were performed for * K* = {100, 200, 500, 1000, 2000} long records over 1000 Monte Carlo repetitions.

#### GCT performance

**Fig. 10:**
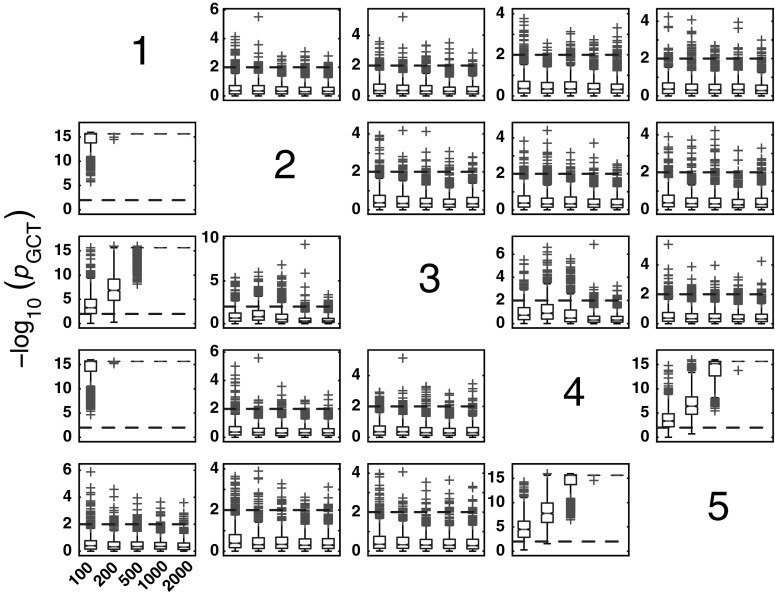
GCT performance for Model 2 and * K* = {100, 200, 500, 1000, 2000} data record lengths

As before, Model 2 also shows that GCT’s performance improves with the increased record length (Fig. 10). At * K* = 200, GCT already performs well with FN rate below 5 %, reaching overall FN rates below 2 % for * K* = 2000.

#### *i*PDC performance

**Fig. 11:**
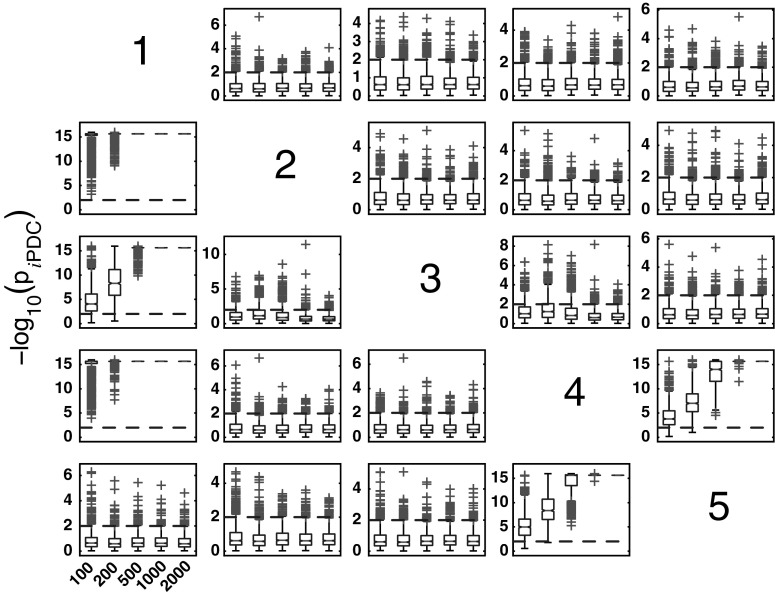
*i*PDC performance for Model 2 and * K* = {100, 200, 500, 1000, 2000} data record lengths

**Fig. 12:**
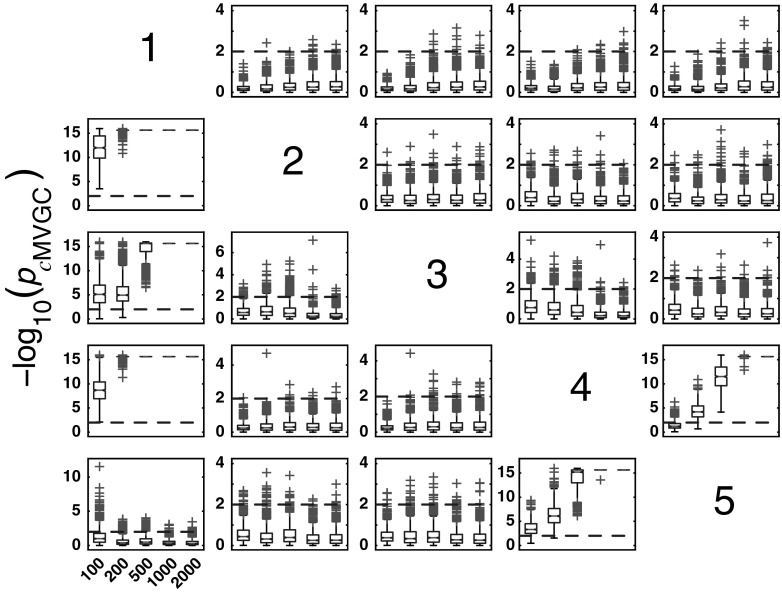
*c*MVGC performance for Model 2 and * K* = {100, 200, 500, 1000, 2000} data record lengths

**Fig. 13:**
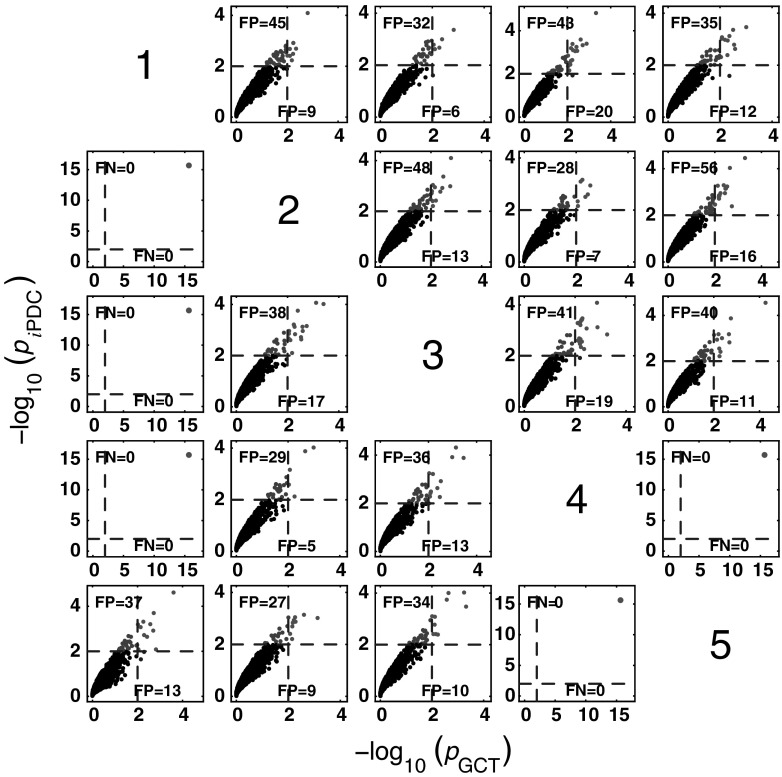
Comparative performance between GCT and *i*PDC detection performances at * K* = 2000 time samples for Model 2

For Model 2, FN rates are practically negligible when *K* > 200 for all measures of GCT, *i*PDC, and *c*MVGC (See Figs. 10–12). Overall, the pattern of *i*PDC performance is similar to that of GCT’s. Yet *i*PDC’s FP rates are slightly higher than GCT’s. For example, performance for *K* = 2000 is between 2.7 and 5.6 % (Fig. 13).

#### *c*MVGC asymptotic behaviour for Model 2

**Fig. 14:**
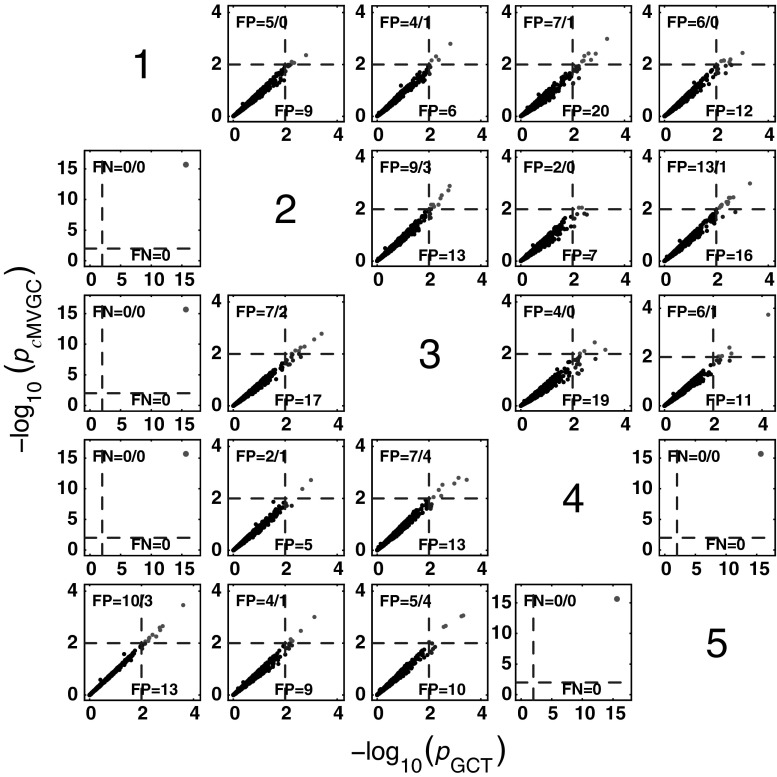
Comparative performance between GCT and *c*MVGC detection performances at * K* = 2000 time samples for Model 2

*c*MVGC performance for * K* = {100, 200, 500, 1000, 2000} is shown in Fig. 12. When taken with respect to GCT (Fig.  14), FPs are consistently lower than GCT’s for K = 2000 and, as in the case of the previous model, it does not conform to a preset $\alpha =1\,\%$ for FP rates. This is also easy to appreciate for other values of $K$ in Fig. 12 as most outliers (red crosses) are below the −log_10_(*p* value) = 2 line for nonexisting connections.

Taking GCT as a reference, trial-by-trial comparisons of *i*PDC and *c*MVGC, respectively, confirm the pattern of higher FP for the former compared to a pattern of FP, below 1 %, for *c*MVGC with or without correction (See Figs. 13, 14). This is also suggestive of possible problems encountered in how the MVGC package handles the FP rate, which may be fortuitously benign to MVGC in this example, but does not represent the general case, since it does not hold for Model 1.

### Model 3: Modified five-var model

**Fig. 15:**
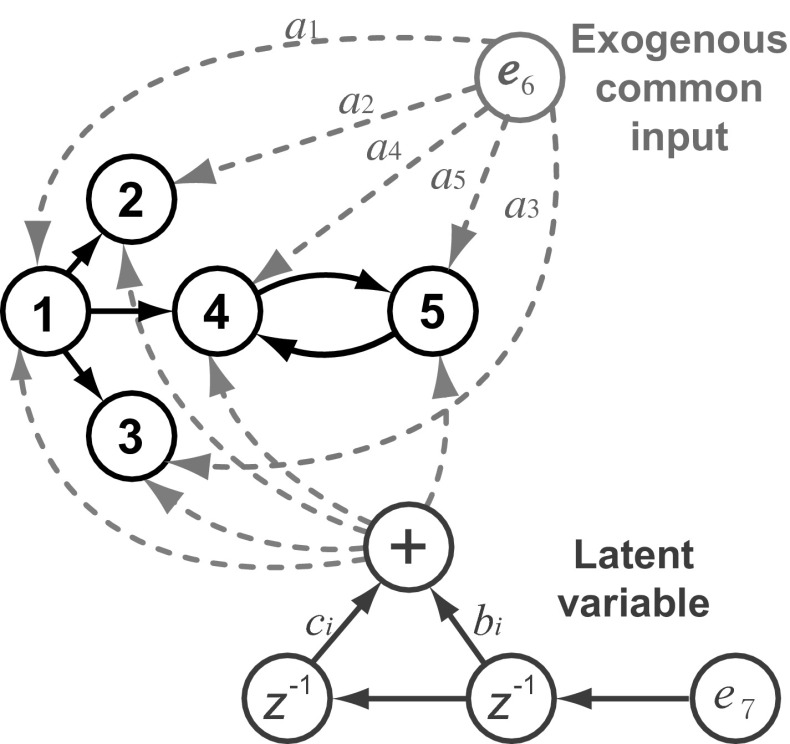
Diagram depicting the essential elements of model introduced by [19] modified from Model 2 [6]. For each simulation, the parameters $a_i$ were chosen randomly from a uniform distribution in $[0\; 1]$ interval, and all $b_i=2$ and $c_i=5$, while the innovations, $e_i$, were drawn from random variables with ${\mathcal{N}}\{0,1\}$

To further probe the statistical behaviours of GCT, *i*PDC and *c*MVGC, we simulated the modified five-channel toy Model 3, originally introduced in [6], under the formulation variant proposed by [19] and reproduced here for reference (Fig. 15).

The corresponding set of equations is

3 $$\begin{aligned} \left\{ \begin{aligned} x_{1}(t) &=\ 0.95\sqrt{2}x_{1}(t-1) - 0.9025x_{1}(t-2) \\&\quad +\, e_{1}(t) + a_1 e_{6}(t)+ b_1 e_{7}(t-1) + c_1 e_{7}(t-2) \\ x_{2}(t) &=\ 0.5x_{1}(t-2) \\&\quad+\, e_{2}(t) + a_2 e_{6}(t) + b_2 e_{7}(t-1)+ c_2 e_{7}(t-2) \\ x_{3}(t) &=-0.4x_{1}(t-3) \\&\quad +\, e_{3}(t)+ a_3 e_{6}(t) + b_3 e_{7}(t-1)+ c_3 e_{7}(t-2) \\ x_{4}(t) &=-0.5x_{1}(t-2) + 0.25\sqrt{2}x_{4}(t-1) + 0.25\sqrt{2}x_{5}(t-1)\\&\quad+\, e_{4}(t)+ a_4 e_{6}(t) + b_4 e_{7}(t-1) + c_4 e_{7}(t-2) \\ x_{5}(t) &=-0.25\sqrt{2}x_{4}(t-1) + 0.25\sqrt{2}x_{5}(t-1) \\&\quad+\, e_{5}(t) + a_5 e_{6}(t)+ b_5 e_{7}(t-1) + c_5 e_{7}(t-2), \end{aligned} \right. \end{aligned}$$

additionally containing the large exogenous input $e_6(t)$ and the latent variable $e_7(t)$. In the simulations, $e_i(t)$ were uncorrelated zero mean unit variance Gaussian innovation noises, and the parameters were chosen as $a_i \sim U(0,1),\, b_i = 2$ and $c_i = 5, i = 1, \dots ,5$ according to [19].

The proposal in [19] of introducing exogenous/latent variables is an interesting idea which allows investigating the influence of large common additive noise sources on the performance of GCT, *i*PDC and *c*MVGC. Here, to assess the impairment that the extra exogenous/latent variables possibly inflict on null-hypothesis testing, we repeated the procedure not just under the same conditions of [19], but also using a broader range of data record sizes:

$$\begin{aligned} K=\{100,\,200,\,500,\,1000,\,2000,\,5000,\,10000\}. \end{aligned}$$

#### GCT performance in the presence of exogenous noise, Model 3

**Fig. 16:**
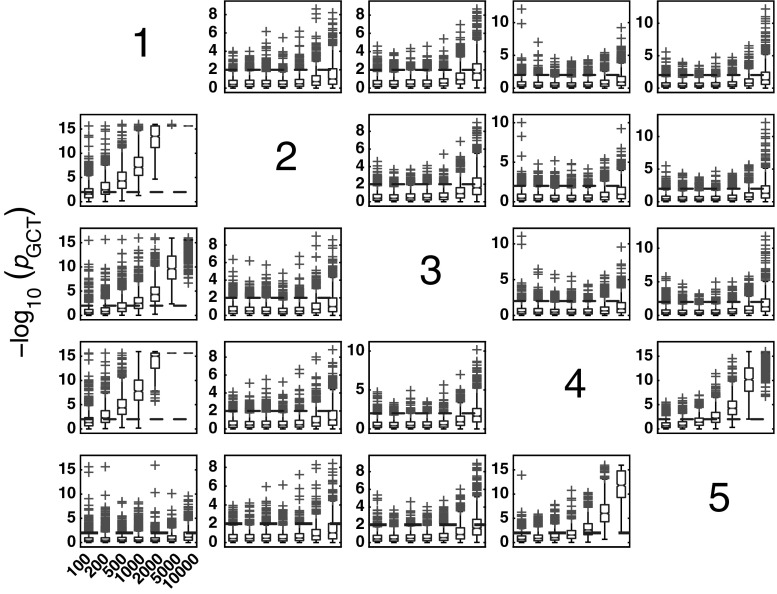
GCT performance on Model 3 and * K* = {100, 200, 500, 1000, 2000, 5000, 10000} data record lengths

The GCT performance for Model 3 can be appreciated in Fig. 16. When compared with Model 2, GCT’s performance deteriorates in the presence of exogenous noises. Interestingly, its performance with respect to detecting existing connections increases with longer data records, while in the absence of connections, the FP rates increase sharply especially for the * K* = 10000 case. For * K* = 500, the overall FP rates are between 2.3 and 7.9 % with a median of 3.8 %. At $K=10000$, the latter rates grow to a range between 20.8 and 40.4 % with the median value of $26.6\,\%$. FN rates are negligible.

#### *i*PDC performance in the presence of exogenous noise

**Fig. 17:**
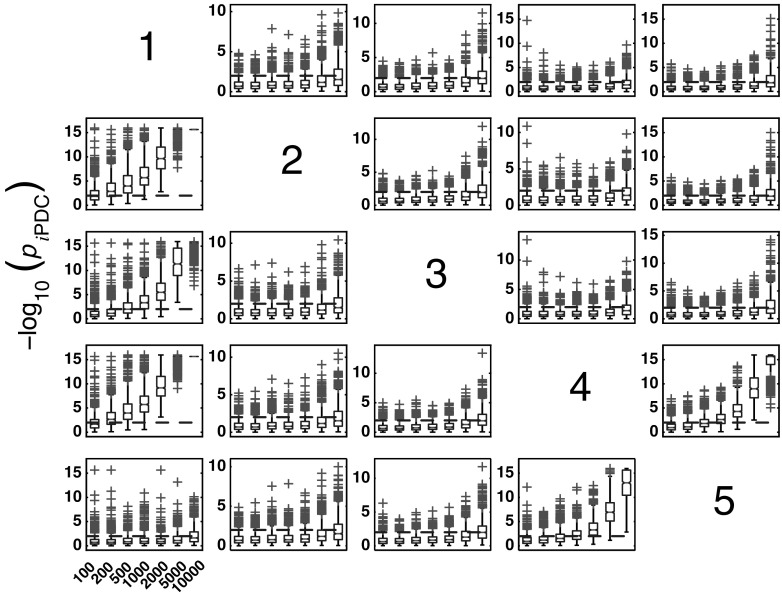
*i*PDC performance on Model 3 and * K* = {100, 200, 500, 1000, 2000, 5000, 10000} data record lengths

*i*PDC performance in detecting connectivity is similar to GCT’s (See Figs. 16, 17). As noted before, *i*PDC tends to have higher FP rates compared with GCT due possibly to the chosen frequency domain detection criterion of using a single-frequency with significant * p* value as indicative of a valid connection. Overall, FP rates range between 6.7 and 11.7 % (median $8.5\,\%$) at $K=100$ increasing to the range $(30.8,49.6\,\%)$ range (median $40.1\,\%$) at * K* = 10000.

#### *c*MVGC performance for Model 3

**Fig. 18:**
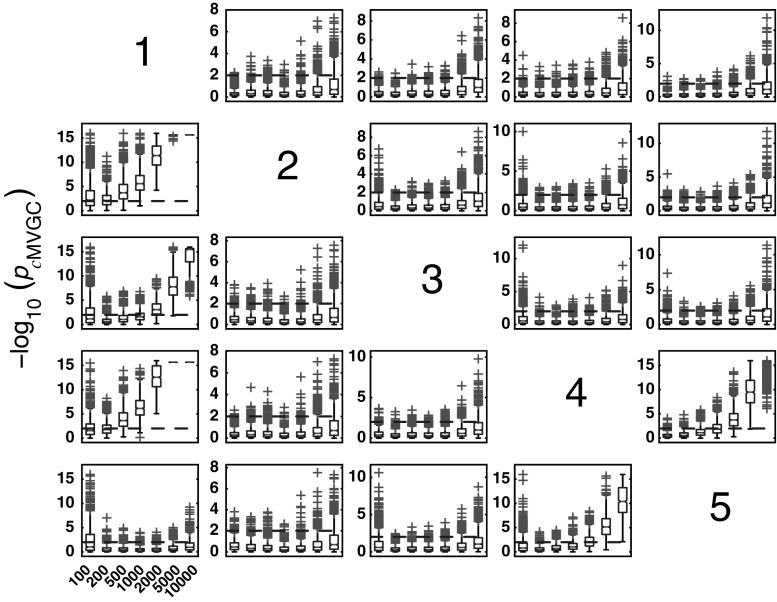
*c*MVGC performance on Model 3 and * K* = {100, 200, 500, 1000, 2000, 5000, 10000} data record lengths

**Fig. 19:**
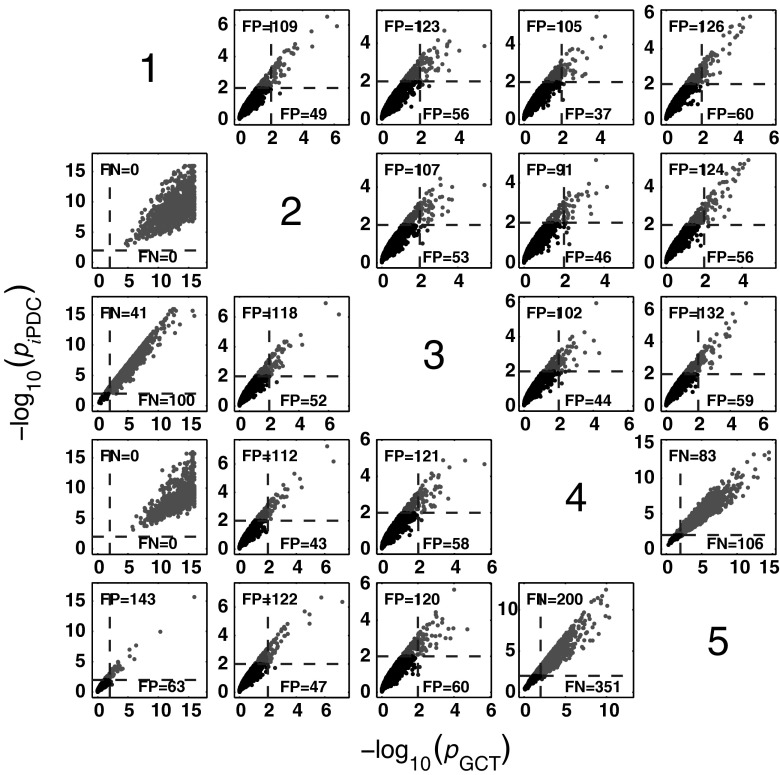
Comparative performance between GCT and *i*PDC detection performances at $K=2000$ time samples for Model 3

**Fig. 20:**
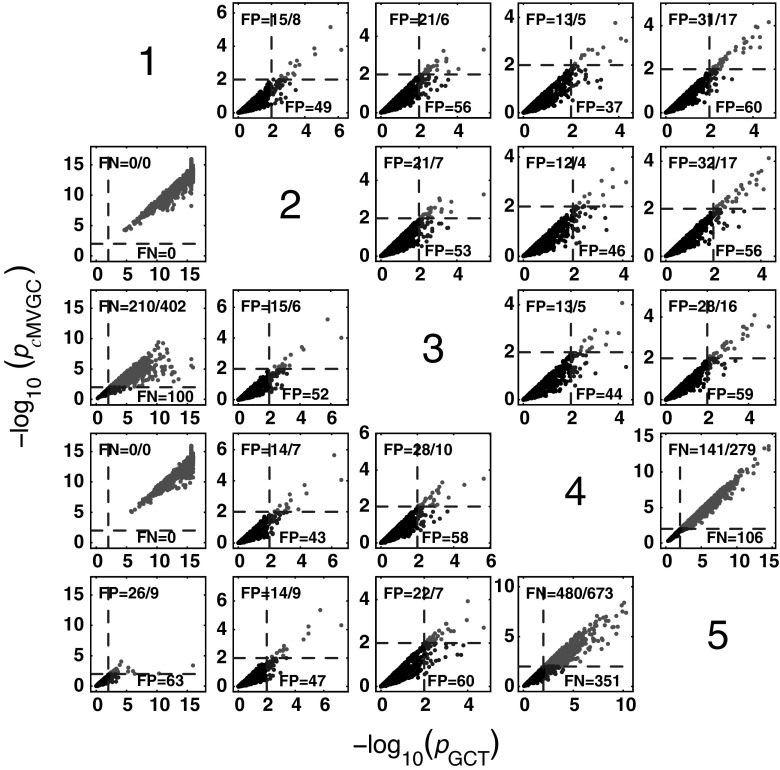
Comparative performance between GCT and *c*MVGC detection performances at $K=2000$ time samples for Model 3

Here (Fig.  18) the qualitative behaviour is the same as for the other estimators. However, as for Model 1, false decision rates are out of control,—sometimes, much below GCT’s, and sometimes, way above it, irrespective of corrections which fail to restore Neyman–Pearson expected behaviour. Again taking GCT as reference, Fig. 19 shows the similarity of iPDC’s result to GCT’s with the same pattern of larger FP values well above α=1%. The corresponding results for cMVGC compared with GCT portray a bias towards lower cMVGC FPs (Fig. 20).

## Discussion

This study presents simulation evidence about the performances of statistical connectivity tests: two in time domain and using two new frequency domain measures.

One should remind the reader that the frequency domain tests, *i*DTF and *i*PDC, measure different aspects of connectivity and are not immediately comparable as discussed at length in [17, 18]. This contrasts with GCT, *i*PDC and *c*MVGC which are geared towards describing the same aspect of connectivity between adjacent structures [17]. Among the tests in the latter class, GCT proved to be the one most in accord with the expected Neyman–Pearson behaviour in the sense that observed FP rates are in accord with the preset value of $\alpha$ justifying its employment as reference in the trial-by-trial comparisons between methods as summed up herein.

Qualitatively *i*PDC closely mirrors GCT behaviour, and predictably produces higher FP rates as a consequence of how *i*PDC connectivity was detected by deeming just one frequency above threshold as significant. Whereas one may conceivably improve on how to employ *i*PDC for testing, its use is recommended when there is frequency content of physiological interest.

Added for comparison, *c*MVGC detection proved to be biased towards a reduction of the FP rates in many cases. By contrast, examination of its behaviour for other $K$ (available in more detail from our Web site) suggests that, for small $K$, it tends to miss existing connections more often than the other methods.

Perhaps more striking and more important, however, in the sense of Neyman–Pearson detection for $\alpha$ compliance, is that procedures are usually constructed to impart control over FP decisions, which according to the present observations, is a condition that fails to be met by the *c*MVGC implementation from [9] which was used here without modification. It is also important to note that employing author-recommended decision corrections [9] usually aggravates matters. It is this lack of compliance to Neyman–Pearson criteria that we termed *anomalous*. Whether this happens due to an eventual software glitch, or reflects a more fundamental issue, is unknown. One should note that on many instances, *c*MVGC produced fewer FPs, something good in itself. This apparent quality is counterbalanced by much worse performance for some links, as in Model 1, in sharp contrast to other methods whose results attain the prescribed $\alpha$ and are balanced for all connections to within the attainable accuracies of the Monte Carlo simulations.

Based on its good asymptotic control of FP observations, it is fair to suggest that, at least provisionally, GCT, as proposed by [2], be taken as a gold standard for detecting connectivity between adjacent structures and that *i*PDC and *c*MVGC should be used taking into account adequate forewarning of their present observed limitations.

The present Monte Carlo simulations showed good large sample fit and robustness for Models 1 and 2. In the presence of large exogenous/latent variables (Model 3), we observed poor performance for large samples possibly due to the poor performance of the MAR model estimation algorithms under low signal-to-noise ratio regardless of the statistical procedure ($K>5000$). In this regard, Model 3 deserves the special comment that its comparatively worse performance is not surprising since, strictly speaking, it violates the usual assumptions behind the development of all the test detection procedures discussed herein.

Finally, we propose that the present methodology represents the seed of a potential tool for systematically comparing connectivity estimators. The reason for this is twofold: (a) the framework provides a standardized approach whereby comparisons can be made systematically and (b) may be used even in the absence of formally rigorous statistical criteria, i.e. even if only ad hoc decision rules are available and is therefore not restricted to methods with theoretically well-established detection criteria. We have future plans to include bootstrap-based connectivity detection schemes under the same standardized framework for comparison purposes.
